# A spatial decision making framework using neutrosophic VIKOR for wind energy investment in Turkey

**DOI:** 10.1038/s41598-025-18799-w

**Published:** 2025-09-29

**Authors:** Hasan Eroğlu

**Affiliations:** Department of Electrical and Electronics Engineering, Faculty of Engineering and Architecture, Recep Tayyip Erdoğan University, Zihni Derin Campus, 53100 Rize, Turkey

**Keywords:** Renewable energy planning, Wind energy investment, Multi-Criteria decision making (MCDM), Neutrosophic VIKOR, Geographic information systems (GIS), Environmental impact, Wind energy

## Abstract

The growing demand for clean energy and the urgency of reducing carbon emissions have made wind power a key element of Turkey’s renewable energy strategy. However, identifying optimal regions for wind energy investment remains a complex task due to the interplay of technical, spatial, and economic factors, all of which are characterized by varying degrees of uncertainty. Although GIS-based site selection and multi-criteria decision-making (MCDM) methods are widely used, few approaches integrate expert judgment and spatial analysis within an uncertainty-aware national planning framework. This study proposes a novel investment prioritization model that combines Geographic Information Systems (GIS) with the Neutrosophic-VIKOR method to assess regional wind energy potential in Turkey. The model considers five core criteria: wind potential, land cost, energy consumption based on population density, presence of existing wind farms, and expert judgment. Expert input is represented using Single-Valued Neutrosophic linguistic scales. A similarity-based weighting method is used to determine the relative influence of each expert. The resulting Priority Index (PI) highlights Balıkesir, Çanakkale, and İzmir as the top three investment regions due to their wind characteristics and energy demand. Istanbul and Samsun also rank highly, supported by existing infrastructure and consumption levels. The proposed framework offers a replicable, uncertainty-aware tool for supporting national wind energy planning. By combining expert-based neutrosophic modeling with spatial analysis, the study addresses existing methodological gaps and provides actionable insights for investors and policymakers pursuing efficient and balanced renewable energy development.

## Introduction

The growing emphasis on clean energy has motivated researchers to explore a wide range of new topics. Innovations are being put forward in many fields for less CO^2^ emission. Preferring cleaner energy sources^1–3^, environmental approaches in the transportation of energy^4,5^, more organic and environmentally friendly power plants^6–8^ are among the key strategies. Wind energy stands as one of the most significant sources of clean and sustainable power. Wind energy has grown rapidly worldwide^9^ and is now one of the fastest-growing renewable energy sectors^10,11^.

**Fig. 1:**
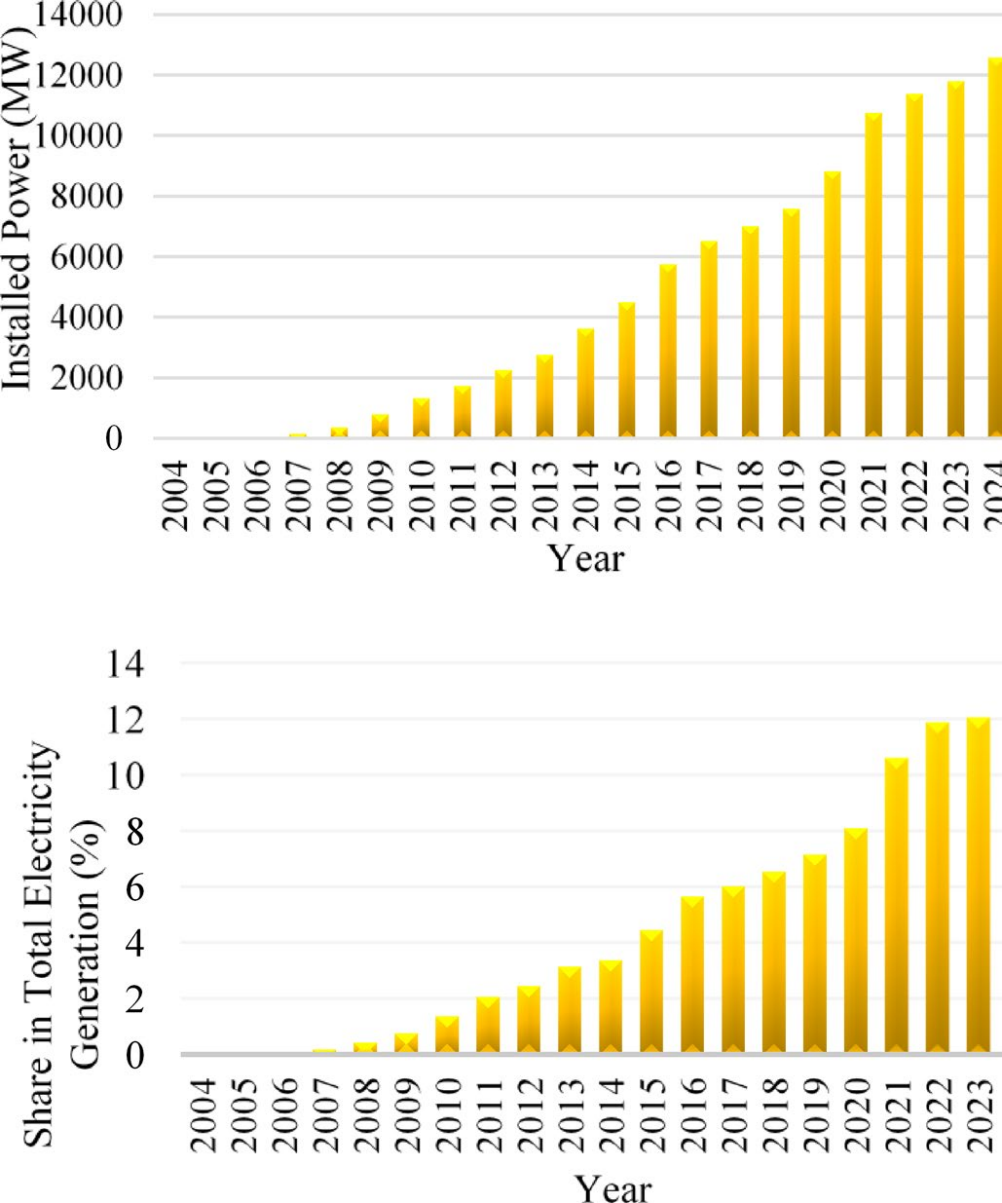
The percentage of wind energy installed power in Turkey in the last 17 years among other energy sources and the change in the amount of installed power^12^.

Parallel to global developments, Turkey has experienced a significant expansion in wind energy capacity over the past 15 years. Important incentives in Turkey’s renewable energy strategy have significantly advanced wind power development. In the background of these incentive policies, there is a search for solutions to important problems such as climate change, electricity supply security, increasing energy prices and access to electricity. The percentage of wind energy installed power in Turkey in the last 15 years among other energy sources and the change in the amount of installed power are as in Fig. 1^12^.

### Literature review on wind farm prioritization

Wind energy potential and the optimal siting of wind farms have been extensively studied in recent years. While many studies have focused on the micro-siting of individual turbines, the development of macro-level planning strategies that incorporate economic, environmental, and technical factors is relatively limited. In Turkey, several GIS-based studies have focused on identifying suitable locations for wind energy development using various MCDM techniques^13–22^. These studies provide valuable insights, but they tend to evaluate individual regions or small scales without offering a broader prioritization framework at the national level.

Beyond Turkey, several notable studies in the Eastern Mediterranean region have addressed similar challenges. In Greece, for instance, Ioannou et al.^23^ developed a comprehensive decision support methodology combining AHP and TOPSIS to evaluate wind farm suitability in the Eastern Macedonia and Thrace region. Their framework accounts for technical, economic, and social criteria and introduces scenario-based flexibility. Likewise, Gazos and Vagiona^24^ analyzed offshore wind suitability in the Thracian Sea using exclusion criteria and zoning strategies supported by GIS and WaSP tools. Together, these studies emphasize the relevance of integrated spatial modeling and underscore the value of multi-scenario planning in regional-scale wind energy strategies.

Several studies have also examined offshore wind farm development in Turkey. For example, Emeksiz and Demirci^17^ applied MCDM models to evaluate offshore locations, while Argin et al.^18^ and Cali et al.^20^, explored techno-economic feasibility. These studies address key spatial planning elements but do not provide a unified investment prioritization mechanism across regions.

From a logistics perspective, Schrotenboer^25^ et al. proposed an Adaptive Large Neighborhood Search Heuristic to optimize technician routing and maintenance schedules in offshore platforms. Their work demonstrates the potential of operational research in extending wind energy efficiency. Further, recent studies by Zhang and Yang^26^, Asadi and Pourhossein^27^, and Verma et al.^28^ have explored wind farm layout, turbulence loss minimization, and capacity optimization through evolutionary algorithms like NSGA-II and genetic algorithms.

On a macro scale, Amjad et al.^29^ introduced a spatial prioritization system for Ghana using clustering, AHP, and density metrics. Such models offer inspiration for integrating socio-economic and technical dimensions but are rarely adapted to national policy frameworks. Moreover, most studies rely on crisp values or fuzzy logic, which limit their ability to account for higher-order uncertainty and vagueness in expert judgment.

In parallel with recent developments in decision-making under uncertainty, several studies have proposed advanced fuzzy and neutrosophic frameworks for renewable energy evaluation. For example, Saeed et al. introduced a robust decision-making model based on fuzzy hypersoft sets integrated with entropy and similarity measures to evaluate and optimize renewable energy resource management in Turkey, addressing the inherent uncertainty in expert-driven assessments^30^. Similarly, Saeed et al. also employed Fermatean Neutrosophic Sets (FNS) to model subjective uncertainty and improve diagnostic decision-making in healthcare, highlighting the adaptability of such models to complex, imprecise systems^31^. Furthermore, Alamri et al. conducted a hybrid entropy-based economic analysis of hydrogen production technologies using multiple MCDM techniques, including VIKOR, TOPSIS, and MultiMOORA, demonstrating the versatility of entropy-weighted methods in prioritizing sustainable energy solutions^32^. In a more recent development, Liu et al. proposed a novel parametric similarity measure for single-valued neutrosophic sets (SVNSs), which improves the reliability of decision-making under ambiguous conditions by allowing flexible parameter adjustment based on decision-makers’ preferences^33^. Additionally, Meng et al. presented a two-stage GIS-based decision model using SVNSs for optimal site selection of waste-to-energy plants in Beijing, emphasizing the value of spatial and neutrosophic integration in addressing uncertainty and imprecision in real-world energy infrastructure planning^34^.

In this context, neutrosophic set theory provides a promising alternative to traditional fuzzy systems by enabling the explicit modeling of truth, indeterminacy, and falsity^35^. Despite its theoretical advantages in modeling uncertainty, the application of neutrosophic logic in renewable energy planning remains limited and underexplored. Few, if any, studies have combined neutrosophic MCDM methods with GIS-based data to construct an investment prioritization index for wind energy at a national scale.

This gap motivates the proposed model, which integrates neutrosophic-VIKOR with GIS-based spatial analysis to develop a Priority Index (PI) for wind energy investment across Turkey. The framework is designed to support policymakers and stakeholders in strategic decision-making, accounting for expert judgment, technical feasibility, and regional policy goals.

Despite the growing body of GIS-based MCDM studies in wind energy planning, there remains a clear methodological gap in national-scale macro-siting models that incorporate higher-order uncertainty. Most existing approaches either focus on local or regional analyses or rely on traditional fuzzy logic, which lacks the capacity to fully represent expert ambiguity. This study addresses this gap by integrating Single-Valued Neutrosophic Sets (SVNS) with the VIKOR method in a GIS environment, offering a novel, uncertainty-aware framework for prioritizing wind energy investments across Turkey.

### Motivation of the study

The strategic siting of renewable energy power plants plays a crucial role in optimizing energy production and ensuring sustainable development. In the literature, two fundamental approaches have been defined for site selection: macro-siting and micro-siting^36,37^. While macro-siting focuses on large-scale factors such as regional wind potential, energy demand, and economic feasibility, micro-siting delves into more localized criteria, including proximity to infrastructure, environmental impact, and land constraints^38,39^. Existing studies have predominantly concentrated on micro-siting analyses, assessing specific locations based on narrowly defined criteria. However, a significant gap remains in research that provides a systematic framework for prioritizing investment regions at a national scale, which is essential for guiding policymakers and investors in wind energy development. The urgency of addressing climate change, coupled with concerns over energy security and rising electricity costs, has intensified the global push for renewable energy solutions. Many governments have introduced incentive policies to accelerate wind power adoption. However, these policies also present challenges, particularly in determining the most suitable regions for new wind power plant installations. Investors must navigate complex decision-making landscapes shaped by regional variations in wind resources, energy needs, land values, and policy environments.

This study aims to tackle these challenges by conducting a comprehensive analysis of Turkey’s wind farm distribution, energy consumption profile, and wind potential. By integrating the *Neutrosophic-VIKOR* method—a robust multi-criteria decision-making approach—with expert evaluations, this research introduces a novel *Priority Investment Index* for wind power plants. This index provides investors with a data-driven tool for identifying high-potential investment regions, thereby enhancing strategic decision-making.

The findings reveal that Balıkesir, Çanakkale, and İzmir emerge as the top three priority cities for wind power investment in Turkey, owing to their high wind energy potential and substantial energy consumption. Beyond offering empirical insights, this study bridges the gap between theoretical frameworks and real-world investment strategies, providing actionable recommendations for policymakers and industry stakeholders. Ultimately, the study enhances data-driven investment strategies and supports Turkey’s ongoing transition to a low-carbon, resilient energy infrastructure.

The remainder of this study is structured as follows: Sect. 2 introduces the study area and data sources. Section 3 outlines the decision criteria and presents the integrated methodology, including Neutrosophic-VIKOR modeling and GIS-based spatial analysis. Section 4 presents the results and spatial prioritization findings, followed by Sect. 5, which provides detailed discussion and theoretical, analytical, and policy implications. Finally, Sect. 6 concludes the study and outlines future research directions.

## The study area

**Fig. 2:**
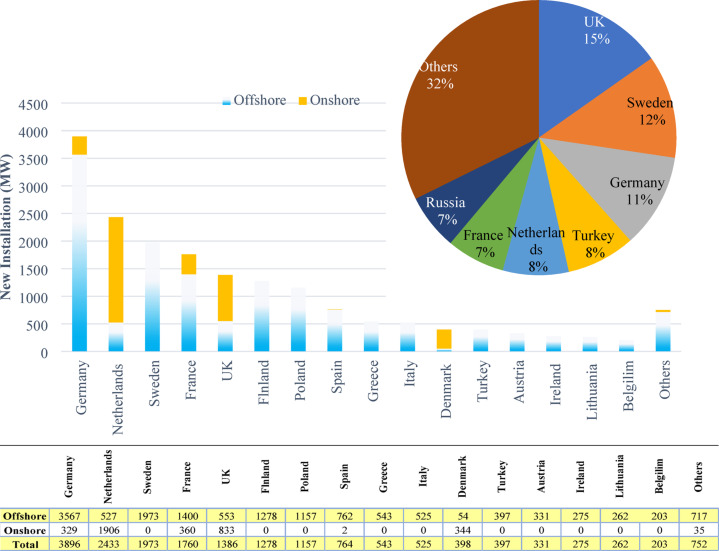
Wind power plant installations among European countries in 2024.

According to data from the General Directorate of Renewable Energy and the General Directorate of State Meteorology Affairs, the wind energy potential of Turkey is estimated to be 48,000 MW. It is in an important place in terms of wind potential among European countries. The country’s administration has significantly increased the wind generation capacity of the country with the incentives related to the wind potential in recent years. The place of Turkey’s new wind installations among other European countries in 2024 is shown in the Fig. 2^40^. According to these values, Turkey is the twelfth country that built the most wind farms in Europe in 2024.

Turkey holds a strategic advantage in terms of renewable energy sources due to its geopolitical position. In line with the country’s development over the years, renewable energy investments have been increased to meet energy demands, achieve low carbon emission targets, and reduce energy costs for consumers. The advantages such as the use of wind energy at night and the less CO^2^ release of wind power plants compared to solar panels are among the prominent advantages of wind energy compared to solar energy.

**Fig. 3:**
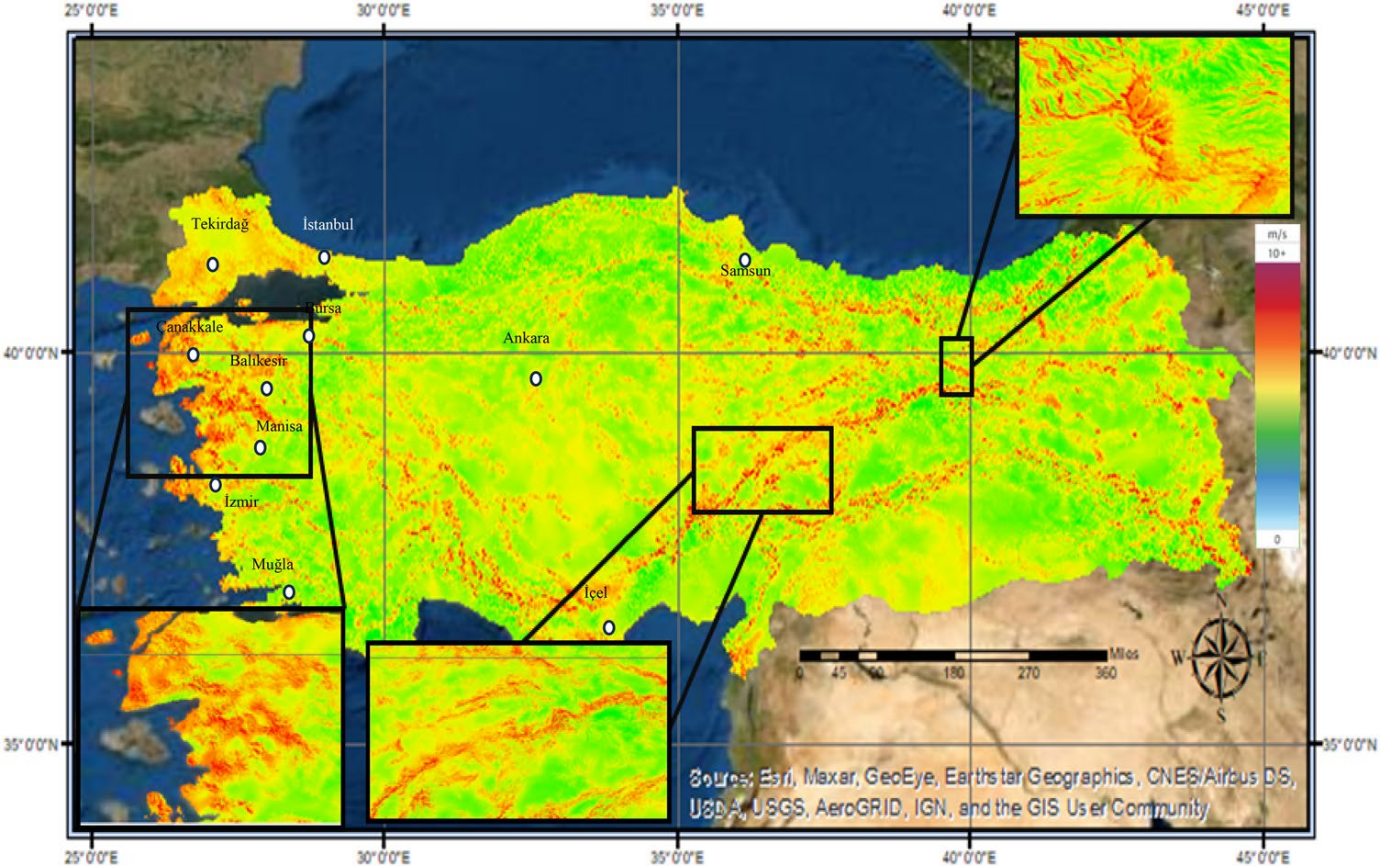
Wind speed map of Turkey.

Considering the overall wind generation potential of the country, it is evident that the western regions of Turkey are particularly advantageous in terms of wind potential. According to Global Wind Atlas data^40^, the wind speed map for Turkey at an altitude of 50 m is shown in the Fig. 3. The areas shown in red tones in Fig. 3 are suitable areas in terms of wind potential. The word suitable used in this study means that the wind potential is at a sufficient level. The basis of this evaluation is the information in the literature that wind farm installation can be economical in regions with wind speed of 7 m/s and above^41–43^. When we look at the country in general, it is seen that the wind generation potential of the country is especially high. However, it is observed that there are areas with significant wind potential in the rest of the country.

The top 5 sources of electricity production in Turkey are 34.8% coal, 25.6% hydroelectric, 22.7% natural gas, 8.1% wind and 3.8% solar energy. Population density and industrialization in Turkey are more concentrated in the western parts of the country. This concentration in trade, industry, and population explains the western region’s energy demand. However, hydroelectric power plants with a significant energy generation potential in the country are in the east of the country^44^. This energy dependence between the east and west of the country brings with it a serious excess in the distances of energy transmission lines. In addition, significant energy distribution losses occur with increasing power transmission line distances. Significant advantages are obtained with the balanced distribution of power generation facilities. These are the decrease in power loss, increase in energy efficiency, security of electricity supply, reduction of operating costs, minimization of environmental damage, reduction of fossil fuel consumption, and reduction of greenhouse gas emissions. Transmission distribution losses in Turkey were averagely 12% between 2005 and 2018^45^. In countries with developed electrical infrastructure, this value is around 6%. These findings highlight the importance of developing a balanced distribution strategy for new power plants.

## Methodology

**Fig. 4:**
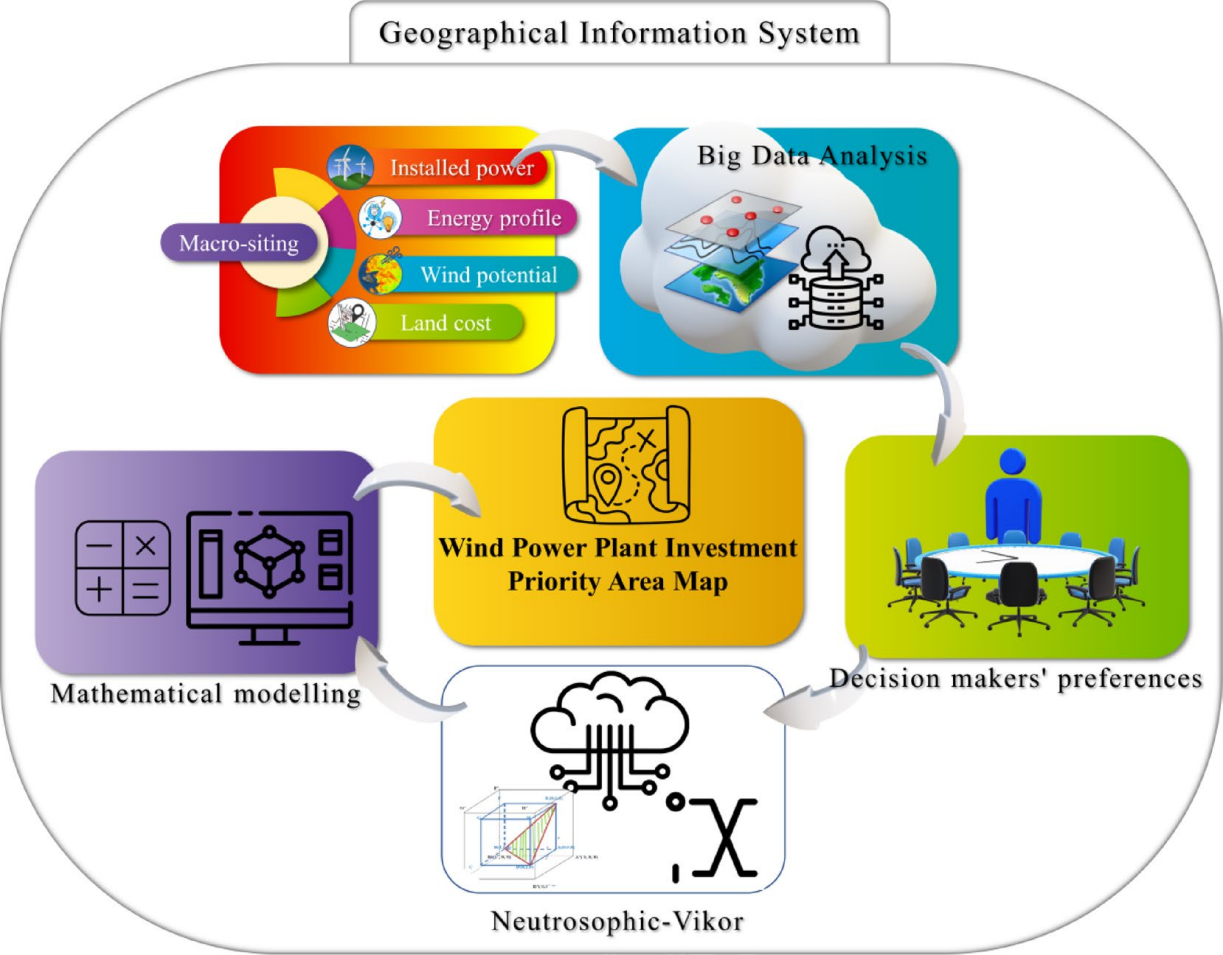
GIS and neutrosophic-Vikor based framework of the study.

In this section, studies have been carried out to determine the investment area priority index, which is an important indicator for wind power plants. First, investment criteria for wind power plants in macro-siting were analyzed. For this purpose, four main parameters were determined. These parameters and their general definitions are given in detail in the following sub-sections. Big data belonging to these three criteria are integrated into Geographical information systems (GIS). For wind farms, the need index value is proposed mathematically by using these data. Wind power plant investment priority area map for Turkey was obtained by processing the data in geographic information systems according to the proposed index value. The obtained findings were examined, and their accuracy was discussed. The framework proposed in the study is presented in Fig. 4.

### Data collection and preprocessing

All datasets used in this study were collected for the year 2024, ensuring temporal consistency across variables. The land cost data were obtained from the Turkish Land Registry and Cadastre Information System (TAKBİS) and processed via the Tapusor platform, which provides average land sales values at the provincial (NUTS-3) level. These values were normalized and transformed into land cost coefficients using GIS-based spatial interpolation techniques. Although more granular data (e.g., district or parcel level) are available, provincial aggregation was preferred to align with the macro-siting scope of the study and to ensure compatibility with other national datasets. Similarly, wind turbine data—including the number, location, and installed capacity of turbines—were sourced from the Turkish Energy Atlas and TEİAŞ. These data were georeferenced and aggregated at the provincial level using GIS tools to generate the installed wind power distribution layer. All spatial datasets were integrated into the GIS environment as vector-based shapefiles rather than raster layers; thus, they do not possess continuous spatial resolution but are instead linked to administrative boundaries. This approach ensures methodological coherence and supports the national-scale investment prioritization objective of the study.

### Macro-siting characteristics

#### Energy demand and population density

One of the most important determining factors in the regionally positioning of a renewable energy source is the population density and energy need of the region^46,47^. There are many advantages of having energy sources close to the regions where energy is consumed intensively. The short distance of energy transmission lines, low line losses, low energy investment and maintenance costs are the main ones.

**Fig. 5:**
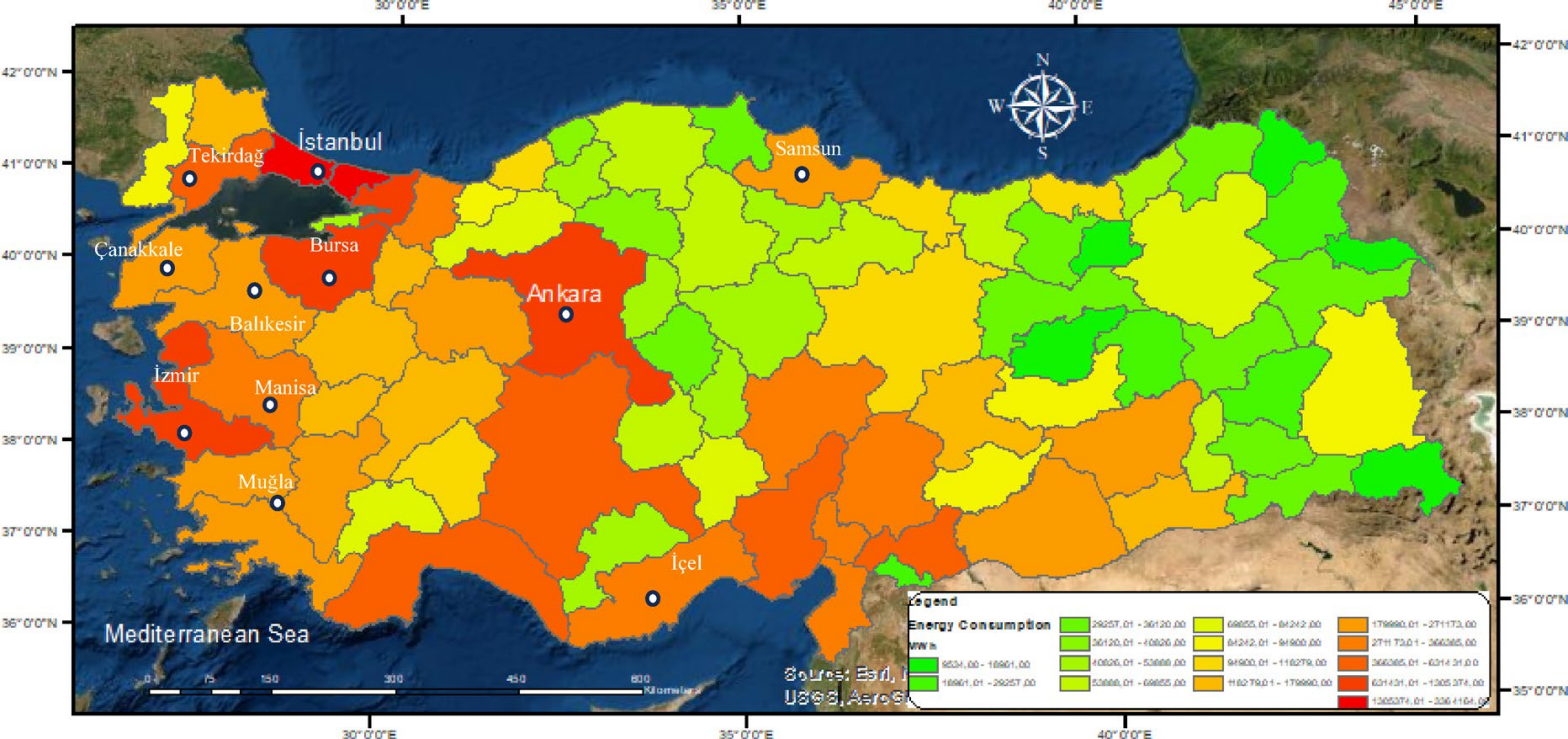
Regional electricity consumption map of Turkey.

In Turkey, major population centers are predominantly located in the western part of the country. Ankara, located in the central region, is the capital and ranks first in terms of both population and energy demand. The energy consumption map presented in Fig. 5 was generated using 2024 province-level electricity consumption data processed through Geographic Information Systems^45^.

#### Wind resource distribution

**Fig. 6:**
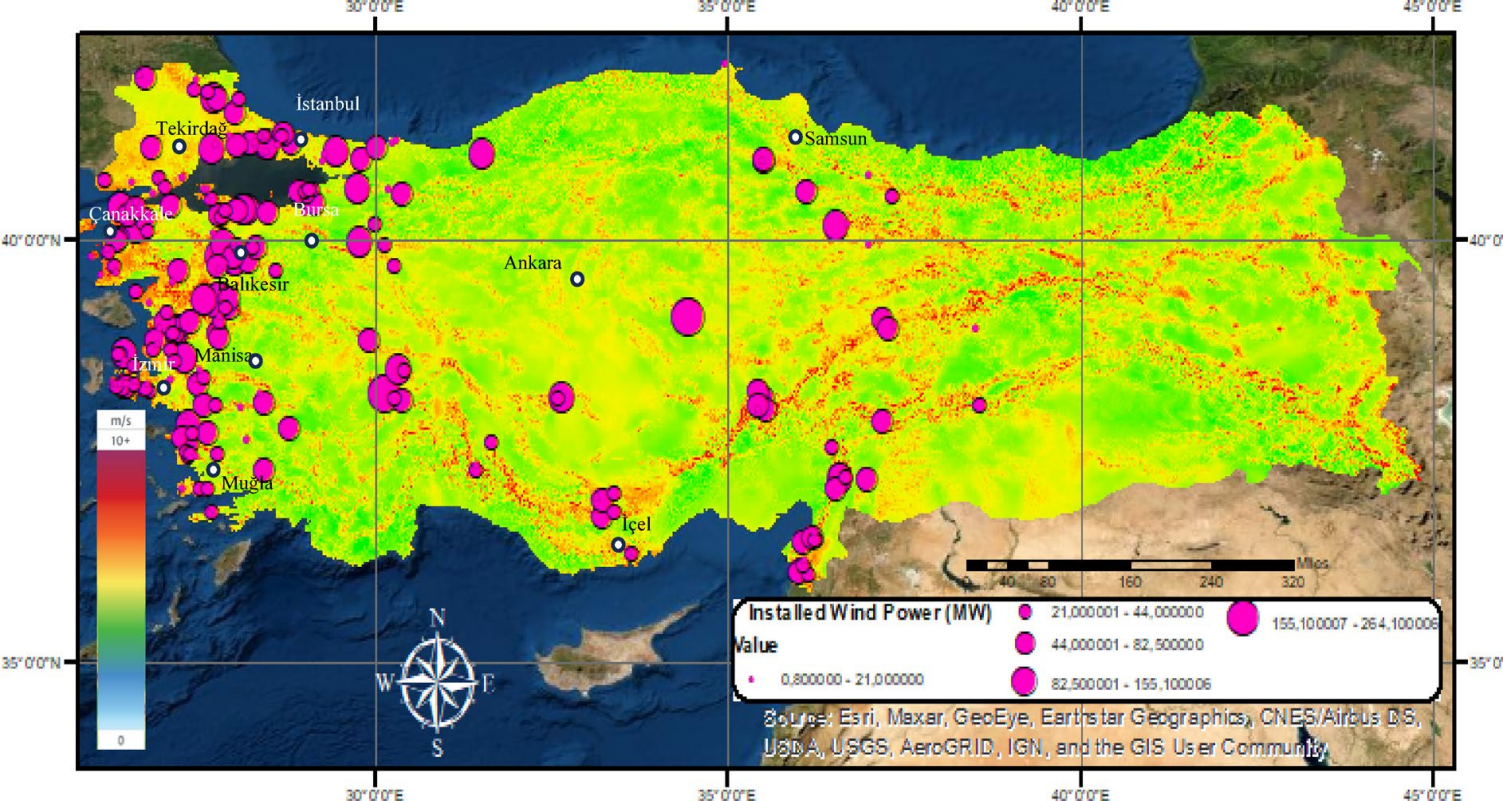
Wind power plant distribution map of Turkey.

As shown in Fig. 3, the high wind potential in western Turkey has resulted in a concentration of wind farms in this region. Using GIS, the geographic locations^48^ and installed capacities of approximately 4.300^49^ wind turbines were analyzed to produce the national wind farm distribution map, as presented in Fig. 6. This map illustrates both the spatial distribution and the installed capacities of wind power plants across the country.

In recent years, with the country’s renewable energy incentive strategies, it seems that wind power plants concentrated in the west of the country have spread to other parts of the country. However, in the country wind energy potential map in Fig. 6, it is seen that wind farms are not established sufficiently in some regions where wind farm installation is suitable in terms of wind potential. With this study, an important need analysis for wind power plants was made considering the energy needs of the country, and the findings were examined in detail in the fourth section.

#### Land cost analysis

The value of land costs is also an important criterion in the macro-siting planning of wind farm installation zones. Land costs may differ from region to region or province to even different neighborhoods. It has been stated in some studies^50^ that expropriation costs for wind farm installation reach 10% of the total project costs. In this context, the land value coefficients of the study area were created for this study by using GIS, artificial intelligence, and Turkey land sales^51^ data.

### Neutrosophic-VIKOR method framework

#### Theoretical foundations

The Neutrosophic VIKOR method is an advanced extension of the VIKOR (VlseKriterijumska Optimizacija I Kompromisno Resenje) method, which is a widely used multi-criteria decision-making technique. This method integrates neutrosophic sets, allowing for a more effective representation of uncertainty, ambiguity, and indeterminacy in the decision-making process. Unlike traditional fuzzy and intuitionistic fuzzy sets, neutrosophic sets consist of three key components: truth membership, indeterminacy membership, and falsity membership functions. This structure provides greater flexibility in handling incomplete, imprecise, and uncertain information, making it particularly useful for complex decision-making scenarios.

The VIKOR method itself is designed to rank and select the best alternative by balancing conflicting criteria. It seeks a compromise solution that minimizes the distance from the ideal solution by determining the alternative with the optimal “S” value, which represents the overall compromise measure. By incorporating neutrosophic sets, the Neutrosophic VIKOR method enhances the traditional VIKOR approach by utilizing neutrosophic linguistic variables and similarity measures, which enable more precise evaluations in uncertain environments.

By combining these concepts, the Neutrosophic VIKOR method offers a more holistic and adaptable decision-making framework. It allows decision-makers to effectively address imprecisions and uncertainties while optimizing solutions across multiple conflicting criteria. This approach is beneficial in complex decision-making environments where ambiguity plays a key role. The following section will provide a detailed explanation of the fundamental principles of neutrosophic sets.

To distinguish our methodology from existing MCDM-GIS approaches in wind energy planning, this study introduces a novel integration of Single-Valued Neutrosophic Sets (SVNS) with the VIKOR method within a spatial GIS-based framework. Unlike traditional fuzzy or crisp MCDM models, the SVNS-based weighting mechanism allows for a more nuanced representation of expert uncertainty by incorporating truth, indeterminacy, and falsity degrees. Furthermore, the similarity-based weighting of decision makers enhances the objectivity of expert aggregation, which is often overlooked in prior studies. This dual-layered integration—SVNS for expert weighting and VIKOR for compromise ranking—within a GIS environment represents a methodological advancement that enables more robust and uncertainty-aware spatial decision-making.

#### SVNS preliminaries

##### Definition 1

^52^ Let D be a Subjective vague and Neutrosophic Set (SVNS) defined in a universe of discourse $\:X=\left\{{x}_{1},{x}_{2},\dots\:,{x}_{n}\right\}$. The set E is characterized by three membership functions: the truth-membership function $\:{T}_{E}\left(x\right)$, the indeterminacy-membership function $\:{I}_{E}\left(x\right)$ and the falsity-membership function $\:{F}_{E}\left(x\right)$. These functions $\:{{T}_{E}\left(x\right),\:{I}_{E}\left(x\right)\:\text{a}\text{n}\text{d}\:\:F}_{E}\left(x\right)$ in $\:X$ are real standard or real non-standard subsets of the open interval $\:\left]{0}^{-},\:\:{1}^{+}\right[$. This means that each function is mapped as follows:

$$\:{T}_{E}\left(x\right):X\to\:\left]\:{0}^{-},\:{1}^{+}\right[$$

$$\:{I}_{E}\left(x\right):X\to\:\left]{0}^{-},\:{1}^{+}\right[$$

$$\:{F}_{E}\left(x\right):X\to\:\left]{0}^{-}\:\:{1}^{+}\right[$$

Furthermore, the sum of the three membership functions satisfies the following conditions:

$$\:{{0}^{-}\le\:sup\:{T}_{A}\left(x\right)+\:sup\:{I}_{E}\left(x\right)}_{E}+\:sup\:{F}_{E}\left(x\right)\le\:{3}^{+}$$

This ensures that the aggregated membership values remain within the extended range of the neutrosophic set, allowing for a more flexible representation of uncertainty, indeterminacy, and falsity in decision-making processes.

$\:{a}_{E}\left(x\right)$, $\:{b}_{E}\left(x\right)$ and $\:{c}_{E}\left(x\right)$ will be used instead of $\:{{T}_{E}\left(x\right),\:{I}_{E}\left(x\right)\:\text{a}\text{n}\text{d}\:\:F}_{E}\left(x\right)\:$in the following.

According to (^35,53^), a single-valued neutrosophic set (SVNS) is described as follows:

##### Definition 2

Let $\:X$ be a universe of discourse. A single valued neutrosophic set $\:E$ over $\:X$ is an object having the form

1 $$\:E=\left\{\langle x,\:\right.\:{a}_{E}\left(x\right),\:{b}_{E}\left(x\right),\:{c}_{E}\left(x\right) \rangle:\:x\:\left. \epsilon \:X\right\}$$.

where the functions.

$$\:{u}_{E}\left(x\right):X\to\:\left[\:0,\:\:1\right],\:{r}_{E}\left(x\right):X\to\:\left[\:0,\:\:1\right],\:{v}_{E}\left(x\right):X\to\:\left[\:0,\:\:1\right]$$

satisfy the constraint:

$$\:0\le\:{u}_{E}\left(x\right)$$+$$\:{r}_{E}\left(x\right)$$ + $$\:{v}_{E}\left(x\right)\le\:3$$ for all $$\:x\in\:X$$.

Here $\:{a}_{E}\left(x\right)$, $\:{b}_{E}\left(x\right)$ and $\:{c}_{E}\left(x\right)$ represent the truth-membership degree, indeterminacy-membership degree, and falsity-membership degree of $\:X$ to $\:E$, respectively.

For simplicity, a SVN number is denoted by $\:E=\left(a,b,c\right),$ where $\:a,b,c$ $\:\epsilon \:\left[\:0,\:\:1\right]$ and $\:0\le\:a+b+c\:\le\:3$.

##### Definition 3

Let $\:{E}_{1}=\left({a}_{1},{b}_{1},{c}_{1}\right)$ and $\:{E}_{2}=\left({a}_{2},{b}_{2},{c}_{2}\right)$ be two SVN numbers, their summation is described as:

2 $$\:{E}_{1}\oplus\:\:{E}_{2}=\left({a}_{1}+{a}_{2}-{a}_{1}{a}_{2},{b}_{1}{b}_{2},{c}_{1}{c}_{2}\right)$$

##### Definition 4

Let $\:{E}_{1}=\left({a}_{1},{b}_{1},{c}_{1}\right)$ and $\:{E}_{2}=\left({a}_{2},{b}_{2},{c}_{2}\right)$ be two SVN numbers, their multiplication is described as:

3 $$\:\:{E}_{1}{\otimes\:E}_{2}=\left({a}_{1}{a}_{2},\:{b}_{1}{+\:b}_{2},\:{c}_{1}+{c}_{2}-{c}_{1}{c}_{2}\right)$$

##### Definition 5

Let $\:E=\left(a,b,c\right)$ be a SVN number and be $\:\lambda\:\epsilon$ℝ an arbitrary positive real number, The scalar multiplication is defined as:

4 $$\:\lambda\:E=\left(1-{\left(1-a\right)}^{\lambda\:},\:{b}^{\lambda\:},\:{c}^{\lambda\:}\right),\:\lambda\:>0$$

##### Definition 6

The complement of a SVNS $\:E$ is denoted by $\:{E}^{c}$ and is described as $\:{a}_{E}^{c}\left(x\right)={\text{c}}_{E}\left(x\right),\:{b}_{E}^{c}\left(x\right)={1-\:\text{b}}_{E}\left(x\right),\:{c}_{E}^{c}\left(x\right)={\:a}_{E}\left(x\right)$ for any x in X. Thus, the complement set is given by:

5 $$E^{c} = \left\{ {x,~~c_{E} \left( x \right),~1 - b_{E} \left( x \right),~~a_{E} \left( x \right)~|x\varepsilon X} \right\}$$

Based on prior studies^35^, weighted aggregation operators related to SVNSs are as follows^54^:

##### Definition 7

Let $\:\left\{{E}_{1},{E}_{2},{E}_{3},\:\dots\:,\:{E}_{n}\right\}$ be the set of $\:n$ SVN numbers, each SVN number is represented as $\:{E}_{j}=\:\left({a}_{j},{b}_{j},{c}_{j}\right)\:(j=\text{1,2},\dots\:,n)$. The single valued neutrosophic weighted average operator as defined in^54^ is given by:

6 $$\:\sum\:_{j=1}^{n}{\lambda\:}_{j}{E}_{j}=\:\left(1-\prod\:_{j=1}^{n}{\left(1-{a}_{j}\right)}^{{\lambda\:}_{j}},\:\left(\prod\:_{j=1}^{n}{\left({b}_{j}\right)}^{{\lambda\:}_{j}},\:\prod\:_{j=1}^{n}{\left({c}_{j}\right)}^{{\lambda\:}_{j}}\right)\right)$$

where $\:{\lambda\:}_{j}$ represents the weight associated with $\:{E}_{j}\:\:\left(j=\text{1,2},3,\dots\:,\:n\right)$, $\:{\lambda\:}_{j}\epsilon\left[\:0,\:\:1\right]$ and $\:\sum\:_{j=1}^{n}{\lambda\:}_{j}=1$.

To date, various score functions have been proposed to rank SVN numbers. One of the most commonly used score functions is as follows:

##### Definition 8

A Single-Valued Neutrosophic (SVN) number is represented as $\:E=\left(a,b,c\right)$ where $\:a,b,c$ $\:\epsilon \:\left[\:0,\:\:1\right]$. The score function for a single-valued neutrosophic number can be calculated as:

7 $$\:S\left(E\right)=\frac{\psi\:a+\phi\:\left(1-b\right)+\zeta\:\left(1-c\right)}{3}$$

where $$\:S\left(E\right)\:\epsilon\left[0,\:\:1\right]$$ and $$\:\psi\:,\phi\:,\zeta\:\:\epsilon\:\left[0,\:\:1\right]$$.

##### Definition 9

Given an SVN number $\:E=\left(a,b,c\right)$ where $\:a,b,c$ $\:\epsilon\:\left[\:0,\:\:1\right]$. The score function exhibits the following characteristics:

1. The minimum score value occurs when $\:\psi\:=0$ and $\:\phi\:=\zeta\:=1,$ representing the most pessimistic scenario.
2. The maximum score value is attained when $\:\psi\:=1$ and$\:\:\phi\:=\zeta\:=0$ corresponding to the most optimistic scenario.

##### Definition 10

For an SVN number $\:E=\left(a,b,c\right)$ where $\:a,b,c$ $\:\epsilon\:\left[\:0,\:\:1\right]$, the accuracy function $\:\epsilon\:\left(E\right)$and the certainty function $\:\gamma\:\left(E\right)\:$ are defined as:

8 $$\:\epsilon\:\left(E\right)=\psi\:a-\zeta\:c$$

9 $$\:\gamma\:\left(E\right)=\psi\:a$$

where $\:\epsilon\:\left(E\right)\:$ and $\:\gamma\:\left(E\right)\:\epsilon\left[0,\:\:1\right]$and $\:\psi\:,\zeta\:\:\epsilon\:\left[0,\:\:1\right]$.

In this study, we adopt the commonly used parameter setting $\:\psi\:=\phi\:=\zeta\:=1$, as recommended by Peng et al.^55^, to ensure a balanced contribution of truth, indeterminacy, and falsity in the score calculation. This configuration maintains neutrality and aligns with standard practices in the neutrosophic decision-making literature.

##### Definition 11

Let $\:E=({E}_{1},{E}_{2},{E}_{3},\:\dots\:,\:{E}_{n})$ and $\:F=({F}_{1},{F}_{2},{F}_{3},\:\dots\:,\:{F}_{n})$ be two vectors of $\:n$ SVN numbers. Each element is defined as:

$\:{A}_{j}=〈{{a}_{j}}^{A},\:\:{{b}_{j}}^{A},\:{{c}_{j}}^{A}〉$, $\:{F}_{j}=〈{{a}_{j}}^{B},\:\:{{b}_{j}}^{B},\:{{c}_{j}}^{B}〉$, for $\:(j=1,\:2,\:\dots\:,\:n)$, The generalized distance metric between these two vectors is given by:

10 $$\:{\tilde{d}}_{G}\left(E,F\right)={\left(\frac{1}{4n}\sum\:_{j=1}^{n}\left(\left({\left|{{a}_{j}}^{E}-{{a}_{j}}^{E}\right|}^{\lambda\:}+{\left|{{b}_{j}}^{E}-{{b}_{j}}^{F}\right|}^{\lambda\:}+\:{\left|{{c}_{j}}^{E}-{{c}_{j}}^{F}\right|}^{\lambda\:}\right)+{\left|S\left({E}_{j}\right)-S\left({F}_{j}\right)\right|}^{\lambda\:}\right)\right)}^{\raisebox{1ex}{$1$}\!\left/\:\!\raisebox{-1ex}{$\lambda\:$}\right.}$$

where it gives Hamming distane for $\:\lambda\:=1$ and Euclidean distance for $\:\lambda\:=2$.

#### Construction of the decision matrix

The VIKOR method, first introduced by Opricovic and Tzeng in 2004, is designed to rank alternatives based on conflicting criteria, ultimately identifying the best compromise solution. This approach aims to balance competing objectives in decision-making. The VIKOR technique utilizes the $\:{L}_{p}$ metric as its core principle for measuring distances between alternatives^56^.

11 $$\:{L}_{pj}={\left(\sum\:_{i=1}^{n}{\left({w}_{i}\frac{{{f}^{*}}_{i}-{f}_{ji}}{{{f}^{*}}_{i}-{{f}^{-}}_{i}}\right)}^{p}\right)}^{\frac{1}{p}}\:,\:1\le\:p\le\:+\infty\:,\:j=\text{1,2},\dots\:,j$$

Where $\:{w}_{i}\left(i=\text{1,2},\dots\:.,I\right)\:$ represents the weight assigned to each attribute, while $\:{{f}^{*}}_{i}={max}_{j}{f}_{ji}$ and $\:{{f}^{-}}_{i}={min}_{j}{f}_{ji}$ denote the best and worst possible values of the attributes, respectively. The computed $\:{L}_{pj}$ distance measures how far an alternative $\:{A}_{i}$ is from the optimal solution. The fundamental idea behind this method is to maximize group benefits while minimizing individual dissatisfaction. The feasible solution $\:{F}^{c}$ is considered the closest to the ideal solution.

In multi-criteria decision-making problems that involve multiple decision makers, it is necessary to compute their respective weights to construct an aggregated decision matrix. This study introduces a new approach for determining decision maker weights using recently developed distance measures based on Subjective Vague and Neutrosophic Sets (SVNSs).

Consider the decision matrix $\:{X}^{T}=\left({x}_{ij}^{t}\right)$, which represents the matrix for the $\:t$ decision maker. This matrix consists of $\:n$ attributes, $\:m$ alternatives, and $\:\varvec{t}$ decision makers. Each element $\:{x}_{ij}^{t}=〈{{a}_{ij}}^{t},\:\:{{b}_{ij}}^{t},\:{{c}_{ij}}^{t}〉$ represents a single-valued neutrosophic (SVN) number.

#### Expert weighting and ideal matrix

##### Definition 12

Given two matrices, $\:X={(x}_{ij})\:$and $\:Y={(y}_{ij}),\:$where $\:{x}_{ij}=〈{{a}_{ij}}^{1},\:\:{{b}_{ij}}^{1},\:{{c}_{ij}}^{1}〉$,$\:{y}_{ij}=〈{{a}_{ij}}^{2},\:\:{{b}_{ij}}^{2},\:{{c}_{ij}}^{2}〉$

The distance measure between them is defined as:

12 $$\:{\tilde{d}}_{G}\left(X,\:Y\right)=\frac{1}{4mn}\sum\:_{i=1}^{m}\sum\:_{j=1}^{n}{\left({\left|{{a}_{ij}}^{1}-{{a}_{ij}}^{2}\right|}^{\lambda\:}+\:{\left|{{b}_{ij}}^{1}-{{b}_{ij}}^{2}\right|}^{\lambda\:}+\:{\left|{{c}_{ij}}^{1}-{{c}_{ij}}^{2}\right|}^{\lambda\:}+{\left|S\left({{x}_{ij}}^{1}\right)-S\left({{x}_{ij}}^{2}\right)\right|}^{\lambda\:}\right)}^{\raisebox{1ex}{$1$}\!\left/\:\!\raisebox{-1ex}{$\lambda\:$}\right.}$$

where $\:S\left({x}_{ij}\right)$ denotes the score function $\:{x}_{ij}$.

To determine the ideal matrix $\:{X}^{*}$ f, the values $\:{(x}_{ij}^{*})$, where $\:{x}_{ij}^{*}=〈{a}_{ij}^{*},\:\:{b}_{ij}^{*},\:{c}_{ij}^{*}〉$ are computed using the average operator, as previously defined in Eq. (6).

Since the importance of each decision maker $\:{\text{D}}_{\text{t}}$ depends on their closeness to the ideal decision, the similarity measure between a decision maker’s matrix $\:{X}^{T}$ and the ideal matrix $\:{X}^{*}$ is defined as follows:

13 $$\:{S(X}^{t},\:{X}^{*})=\frac{{\tilde{d}}_{G}({X}^{t},\:{X}^{c*)}}{{\tilde{d}}_{G}({X}^{t},\:{X}^{*)}+{\tilde{d}}_{G}({X}^{t},\:{X}^{c*)}}$$

where $\:{X}^{c*}$ represents the complement of $\:{X}^{*}$.

Finally, the weight of decision maker $\:{\text{D}}_{\text{t}}$ s given by:

14 $$\:{\delta\:}_{t}=\frac{{S(X}^{t},\:{X}^{*})}{\sum\:_{t=1}^{k}{S(X}^{t},\:{X}^{*})}$$

$$\:\text{w}\text{h}\text{e}\text{r}\text{e}\:{\delta\:}_{t}\ge\:0\:\text{a}\text{n}\text{d}\:\sum\:_{t=1}^{k}{\delta\:}_{t}=1.$$

Thus, the proposed method assigns decision maker weights in the SVN decision-making environment, ensuring a fair and systematic aggregation of multiple expert opinions.

#### Criterion weighting

In decision-making scenarios, different decision makers may prioritize criteria differently. To establish a unified set of criterion weights, it is essential to aggregate the individual weight values assigned by each decision maker.

One widely used approach for determining criterion weights is entropy-based weighting, which has been applied in various decision-making contexts^57^. The entropy method assigns weights based on the variability in criterion values, ensuring an objective assessment without requiring additional subjective input.

A key principle of entropy weighting is that higher entropy values indicate lower information contribution, meaning that criteria with greater uncertainty receive lower weights, while those with greater significance (i.e., more variation) are assigned higher weights.

The process of computing criterion weights using Shannon entropy is outlined as follows:

Let $\:S$ be a score matrix given by

15 $$\:S=\left|\begin{array}{ccc}\begin{array}{cc}{S}_{11}&\:{S}_{12}\\\:{S}_{21}&\:{S}_{22}\end{array}&\:\begin{array}{c}\cdots\:\\\:\dots\:\end{array}&\:\begin{array}{c}{S}_{1n}\\\:{S}_{2n}\end{array}\\\:\begin{array}{cc}⋮&\:⋮\end{array}&\:\ddots\:&\:⋮\\\:\begin{array}{cc}{S}_{m1}&\:{S}_{m2}\end{array}&\:\cdots\:&\:{S}_{mn}\end{array}\right|\:$$.

where $\:{S}_{ij}=S\left({x}_{ij}\right)$ is the score value of $\:{x}_{ij}$.

Then, we normalize the score matrix S as follows:

16 $$\:\overline{S}=\left|\begin{array}{ccc}\begin{array}{cc}{\overline{S}}_{11}&\:{\overline{S}}_{12}\\\:{\overline{S}}_{21}&\:{\overline{S}}_{22}\end{array}&\:\begin{array}{c}\cdots\:\\\:\dots\:\end{array}&\:\begin{array}{c}{\overline{S}}_{1n}\\\:{\overline{S}}_{2n}\end{array}\\\:\begin{array}{cc}⋮&\:⋮\end{array}&\:\ddots\:&\:⋮\\\:\begin{array}{cc}{\overline{S}}_{m1}&\:{\overline{S}}_{m2}\end{array}&\:\cdots\:&\:{\overline{S}}_{mn}\end{array}\right|$$

where $$\:{\overline{S}}_{ij}={S}_{ij}/\sum\:_{i=1}^{m}{S}_{ij}\:\left(i=\text{1,2},\:\dots\:m;j=\text{1,2},\dots\:,n\right)$$.

The weights of attributes can be calculated as below:

17 $$\:{E}_{j}=-\frac{1}{\text{ln}m}\sum\:_{i=1}^{m}{\overline{S}}_{ij}\text{ln}{\overline{S}}_{ij},\:\:\:(\text{j}=1,\:\dots\:,\text{n})$$

18 $$\:{w}_{j}=\frac{1-{E}_{j}}{\sum\:_{j=1}^{n}(1-{E}_{j})}$$

It is easy to show that $\:0\le\:{w}_{j}\le\:1$ and $\:\sum\:_{j=1}^{n}{w}_{j}$ according to the properties of entropy.

## Application of neutrosophic-VIKOR method

### Implementation steps

In this section, the problem of determining priority investment areas for wind power plants is solved using the Neutrophic-VIKOR method proposed in this study. In decision-making problems, decision makers often express their choices using both clear and uncertain values. As a result, linguistic words and sentences can be used as scaling values instead of pure numerical values. Linguistic scales offer greater flexibility and suitability compared to numerical scales, accommodating the inherent complexity and variability of decision-making contexts. Neutrophic linguistic scales, presented in Table 1, were used along with questions directed to the three experts (decision makers).

### Decision maker profiles

The panel of experts consisted of three decision makers selected based on their professional background and domain expertise. Two of the experts hold doctoral degrees in Energy Systems Engineering and have more than 15 years of combined experience in wind energy project development and academic research. The third expert is a senior operations engineer currently employed at a wind power plant, with a master’s degree in electrical Power Engineering and over a decade of field experience. Their qualifications ensured a balanced integration of academic insight and real-world operational knowledge into the decision-making process.

### Weighting process

The decision maker’s weight values, shown in Table 2, were calculated using Microsoft Excel based on the linguistic terms provided in Table 1, as per Eq. 17. In this study, the ideal decision matrix represents a reference point constructed from the aggregated evaluations of all decision-makers. Specifically, the neutrosophic decision matrix for each expert was first formed based on their linguistic judgments. Then, the ideal matrix was derived by applying a weighted aggregation using the similarity-based weights of the experts. This ideal decision serves as a benchmark to evaluate how close or far each expert’s input is from the consensus. The similarity measures between each decision-maker’s evaluation and the ideal matrix were used to compute their respective influence in the weighting process, as shown in Eq. 17. As shown in Table 2, DM3 has the highest weight (0.36), indicating their stronger influence in the decision-making process compared to DM1 and DM2.

**Table 1:** Linguistic terms for the proposed methodology^58^.

Linguistic term	Neutrosophic set
Very low (VL)	(0.10, 0.90,0.90)
Between very low and low	(0.20, 0.75, 0.80)
Low (L)	(0.30, 0.70, 0.70)
Between low and medium	(0.40, 0.65, 0.60)
Medium (M)	(0.50, 0.50, 0.50)
Between medium and high	(0.60, 0.35, 0.40)
High (H)	(0.70, 0.30, 0.30)
Between high and very high	(0.80, 0.25, 0.20)
Very high (VH)	(0.90, 0.10,0.10)

**Table 2:** Weights of the decision makers.

DM	Weights
DM_1_	0.30
DM_2_	0.34
DM_3_	0.36

To calculate the criterion weights, we utilized the improved score matrix. Using Eqs. (15, 16, 17 and 18), we computed the weights of attributes, as presented in Table 3.

**Table 3:** Weights calculated with the proposed framework.

Criteria	Weights
Wind potential	0.619
Energy consumption	0.145
Installed power of wind farms	0.133
Land costs	0.103

### Priority index calculation and mapping

To determine the priority areas for wind farm investment, the framework presented in this study utilizes a priority index that integrates key factors influencing investment decisions. These factors include wind energy potential, energy consumption, existing wind power infrastructure, and land costs. By considering these elements, the framework aims to provide a more comprehensive evaluation of the most viable locations for wind farm development.

The primary tool for identifying priority areas is the Investment Priority Index (PI), as described in Eq. 19. The PI index is calculated based on various criteria, each with its respective weight to reflect its relative importance in the decision-making process. The PI is designed to quantify the potential of a region for future wind energy investments, considering both natural and economic variables.

The variables used in the PI index are crucial for identifying high-priority regions for wind energy investments. This framework was developed by considering factors such as energy demand, existing wind power plant status, land cost, and the surveys conducted in this study.

19 $$\:\:{PI}_{i}=\frac{\left(\left({P}_{i}\times\:{Pw}_{i}\right)\:+{\left({E}_{i}\times\:{Ew}_{i}\right)}_{i}\right)-\left(\left({L}_{i}\times\:{Lw}_{i}\right)\:+{\left({W}_{i}\times\:{Ww}_{i}\right)}_{i}\right)}{100}$$

In Eq. 1, $\:{PI}_{i}$ is the priority index of wind power plants, $\:{P}_{i}$ is the wind energy potential percentage, $\:{E}_{i}$ is the energy consumption percentage of $\:i$ *th* city, $\:{W}_{i}$ is the installed wind power percentage of $\:i$ *th* city, $\:{L}_{i}$ is the land cost coefficient percentage of $\:i$ *th* city, $\:i$ is the number of provinces, and $\:{Pw}_{i}$, $\:{Ew}_{i}$…are weights of each variable. According to this index formulation, while higher energy consumption and wind potential increase the need index, existing installed capacity reduces it. In addition, the increase in land prices also decreases the PI index. The high installed capacity in a region negatively affects the preferences of investors as it may cause the region to be excluded from government incentives in terms of wind energy production. For example, Çanakkale is not among the 20 connection regions published in the YEKA (Renewable Energy Resource Areas) RES-3 competitions organized to meet the Wind power installation targets in Turkey^59^. The fact that Çanakkale, which has the highest wind potential in the wind energy potential map, is excluded due to its already high level of installed wind capacity.

**Fig. 7:**
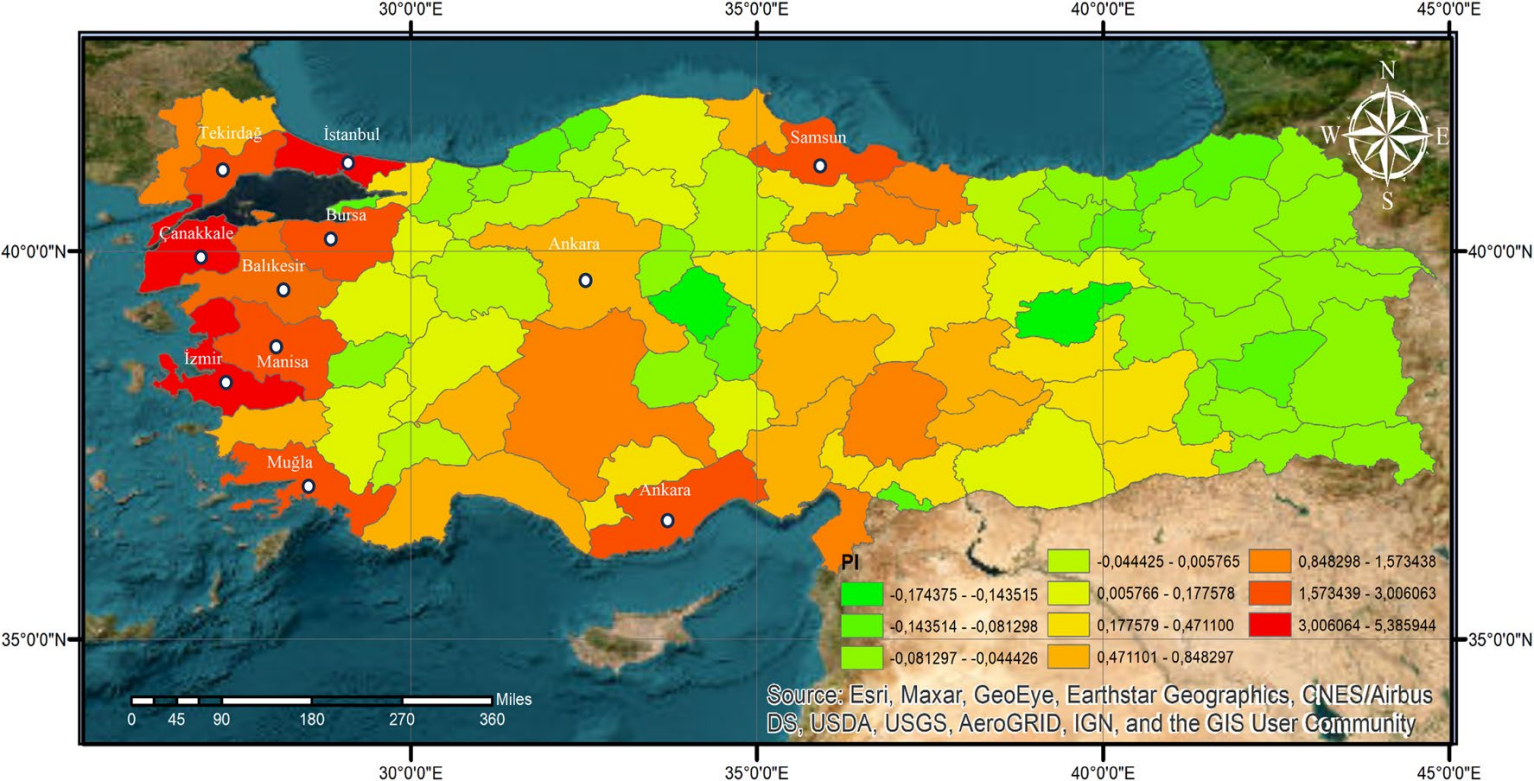
Wind power plant investment priority area map of Turkey.

Using this proposed index, the required data was processed by geographic information systems software, and the wind power plant investment priority area map in Fig. 7 was obtained. This map shows the priority wind farm investment regions of the country by evaluating energy consumption data, installed wind farm data, and wind potential data. The ten priority cities on this map and the PI index values of these cities are presented in Table 4.

**Table 4:** Ranking of the first ten provinces on the wind power plant investment priority area map.

Number	City name	PI
1	Balikesir	5.38
2	Çanakkale	5.05
3	Izmir	4.65
4	Istanbul	3.00
5	Samsun	2.43
6	Tekirdağ	2.22
7	Manisa	2.03
8	Muğla	1.96
9	Bursa	1.95
10	Içel	1.57

## Discussion and accuracy analysis

According to Table 4, Balıkesir holds the highest wind power investment priority. This ranking is consistent with its high wind potential and existing installed capacity, making it an attractive region for investors. Çanakkale follows in second place, benefiting from significant wind resources and strategic location. İzmir, ranked third, stands out due to its high energy consumption, strong industrial infrastructure, and well-developed wind energy sector.

Despite being Turkey’s most populous city and having the highest energy consumption, Istanbul ranks fourth in the wind energy investment priority list with a PI value of 3. This ranking is primarily due to the city’s relatively lower wind potential compared to other regions such as Balıkesir or Çanakkale. Istanbul has relatively limited wind potential compared to other resource-rich cities. However, its significant energy consumption, well-developed infrastructure, and ongoing wind energy projects make it an attractive area for investment. Notably, the construction of Turkey’s largest single licensed wind farm project, with a capacity of 211 MW, in 2021 further emphasizes Istanbul’s growing role in the wind energy sector. Therefore, despite its lower wind potential, Istanbul’s high energy demand and technological infrastructure present a strong appeal for investors, influencing its position in the ranking.

Samsun, ranked fifth, has emerged as a priority area due to its increasing energy demand and suitable land conditions. Despite its location in the Black Sea region, where wind energy investments have been historically limited, Samsun’s favorable geographical features and energy needs make it a viable option for future investments. Among the mid-tier investment regions, Tekirdağ, Bursa, and Manisa have moderate wind potential and energy demand. These cities have relatively lower installed capacities compared to the top-ranked locations, yet their growing industrial activities and strategic locations position them as emerging candidates for wind energy expansion.

Muğla and İçel (Mersin) offer alternative opportunities for investors with their favorable wind conditions and available land. Although their priority index values are lower, these provinces may serve as supplementary regions for wind development, especially in coastal areas with favorable wind profiles.

Examining the Wind Power Plant Investment Priority Area Map, the western part of Turkey emerges as the primary focus for wind energy investments, aligning with the wind potential map (Fig. 3) and installed power distribution (Fig. 6). Balıkesir, Çanakkale, and İzmir clearly stand out as the top three investment destinations, while Samsun is identified as the most promising location in the Black Sea region. The eastern parts of Turkey, however, appear less suitable for wind farm investments, largely due to lower wind potential and infrastructure constraints. Izmir’s ranking is further supported by the findings of the Izmir Development Agency (IZKA), which highlights that the city has significant potential for wind energy development. According to IZKA’s latest report^60,61^ İzmir has a well-established and clustered wind industry, which could foster continued growth and enable investors to benefit from emerging opportunities in the sector.

Although Balıkesir and Çanakkale have higher wind potential values, their energy consumption levels are not as high as in some other major cities, such as Istanbul. This explains why Istanbul and Samsun show higher investment priority than some other cities with stronger wind potential. Additionally, the existing installed capacity in Balıkesir and Çanakkale has led to a convergence in their investment priority values with Istanbul and İzmir.

Given the limited prior research on Turkey’s wind energy investment priorities, direct comparisons remain challenging. However, the consistency of these results with existing literature, investment trends, and national energy policies reinforces the validity of the proposed index. These findings provide a strong analytical basis for policymakers and investors aiming to optimize Turkey’s wind energy expansion strategy.

Compared to previous GIS-based wind planning studies that primarily rely on conventional MCDM techniques such as AHP, TOPSIS, or fuzzy logic, our approach offers a more comprehensive and uncertainty-resilient framework. The incorporation of SVNS enables the model to handle higher-order vagueness in expert evaluations, while the VIKOR method ensures a balanced compromise solution among conflicting criteria. This hybrid model is further strengthened by its spatial implementation in GIS, allowing for the generation of a spatially explicit index for investment prioritization As such, the proposed framework not only fills a methodological gap in literature but also provides a replicable tool for national-scale wind energy planning under uncertainty.

## Policy and managerial implications

In Turkey, the Renewable Energy Resources Support Mechanism (RERSM) entered into force on January 8, 2011, providing incentives for renewable energy sources based on the type of energy and locality. This framework was detailed by the Energy Market Regulatory Board (EMRB), and since its implementation, the number of RERSM participants and the installed capacity have increased significantly in recent years. RERSM guarantees a feed-in tariff of 7.3 USD cents/kWh for electricity produced from wind power plants. In cases where equipment such as blades, generators, and turbine towers are produced domestically, the support increases to 11 Dollars-cent/kWh^62^. In 2021, renewable energy investors in Turkey made significant investments, amounting to nearly 4.900 megawatts, to benefit from the foreign currency-based RERSM, which had an expiration date of December 31, 2020. Due to disruptions in the supply chain caused by the COVID-19 pandemic, this period was extended to June 30, 2021. By the first five months of 2021, investors had already invested approximately 1.800 megawatts. Additionally, the Ministry of Energy and Natural Resources introduced the YEKA auctions, which serve as a market-based policy mechanism. The YEKA strategy, which has been in use since 2005 and 2011, includes a tariff guarantee mechanism and a pre-license auction model. As of December 2021, applications for the YEKA RES-3 competitions had been submitted. With the YEKA auction model, Turkey has made significant progress in developing local production capacity, disseminating relevant technologies, and establishing a competitive domestic market for low-cost renewable energy.

These national policies^63,64^ have significantly contributed to wind energy growth, yet the PI-based analysis reveals the need for more regionally balanced implementation. As of 2023, renewable energy sources accounted for 55.53% of Turkey’s total installed capacity, with wind energy contributing 10.43% to the country’s total electricity production. However, the investment priority map reveals the need for targeted policy adjustments to ensure the balanced development of wind energy across regions. Table 4 indicate that Balıkesir, Çanakkale, and İzmir continue to lead in wind energy investment due to their high wind potential, energy consumption, and existing infrastructure. However, the priority index (PI) values for regions such as İçel (Mersin) and Samsun suggest that there is significant potential in these areas that could be unlocked with focused incentives. This disparity underscores the importance of regional strategies to optimize the distribution of wind energy projects and reduce transmission losses, which are particularly high in Turkey’s current grid structure.

The Priority Index (PI) results presented in Sect. 4.2 serve as a critical input for shaping regional wind energy policies. For instance, Samsun, İçel (Mersin), and Tekirdağ—which rank among the top 10 in the PI index—exhibit strong investment potential due to their favorable wind conditions, energy demand, and relatively lower saturation of existing wind infrastructure. However, these regions have not been prioritized in recent YEKA (Renewable Energy Resource Area) auctions, indicating a misalignment between technical potential and policy focus.

Recommendations for Policymakers:

- Existing incentives should be revised to encourage investments in moderate PI regions like Samsun, İçel, and Tekirdağ, addressing the regional imbalance in wind energy installations.
- Future YEKA auctions should prioritize these underutilized regions to foster a more balanced distribution of wind energy capacity and improve grid efficiency. Allocating projects in these areas can also stimulate local economies and reduce energy transmission distances.
- Investments in grid infrastructure and storage technologies, especially in moderate PI regions, are necessary to support increased wind energy integration and ensure grid stability.
- Expanding domestic production incentives, as currently applied under YEKA, will not only support the wind energy sector but also foster technological advancements and employment in the renewable energy industry.
- Regional energy needs should be integrated into national renewable energy targets. For instance, aligning regional energy demand with available wind potential can enhance system reliability and efficiency.

These strategic measures, aligned with Turkey’s National Energy Plan targets for 2030 and beyond, will ensure the sustainable growth of the wind energy sector while addressing regional disparities and supporting the country’s transition to a low-carbon economy.

On the other hand, there is no regional strategy for the balanced distribution of wind farms in the country. Investors are encouraged with the Energy Specialized Industrial Zones (ESIZ) regulation, which was implemented in Turkey in 2018^65^. The recent announcement regarding the YEKA GES-2024^66^ solar energy auctions align well with the findings of this study, which emphasize the importance of targeted policy adjustments to balance renewable energy distribution across Turkey’s regions. Just as the study recommends focusing on moderate PI regions for wind energy investments, the YEKA auctions reflect a similar effort to encourage the development of renewable energy in underutilized areas.

The six upcoming solar energy auctions, spread across different provinces, including Antalya, Karaman, Malatya, Van, Kütahya, and Konya, are designed to allocate significant capacity to regions that are not traditionally recognized for their solar potential. This aligns with the study’s recommendation to revisit existing incentive mechanisms and distribute renewable energy projects more evenly across the country. By prioritizing regions such as Samsun, İçel, and Tekirdağ for wind energy, and similarly focusing on diverse areas for solar investments, Turkey can achieve a more balanced and efficient energy mix.

Moreover, the government’s efforts to implement long-term contracts and provide purchase guarantees for solar energy align with the broader policy recommendations in the study, particularly the need for infrastructure development and the enhancement of local content requirements. By integrating such initiatives across both wind and solar energy sectors, Turkey can strengthen its renewable energy capacity and reduce regional disparities in energy distribution, addressing challenges in grid efficiency and energy transmission.

To address this, we recommend that future YEKA tenders explicitly incorporate PI-based regional prioritization to ensure a more balanced and data-driven allocation of wind energy investments. For example, Samsun, with its growing energy demand and underutilized wind potential, could benefit from targeted incentives such as grid infrastructure upgrades and localized feed-in tariffs. Similarly, İçel and Tekirdağ could be included in upcoming YEKA RES competitions to diversify regional development and reduce transmission losses from over-concentrated western regions.

By aligning national incentive mechanisms with the PI index outcomes, policymakers can improve the efficiency, equity, and long-term viability of Turkey’s wind energy development.

Beyond its managerial relevance, this study offers concrete theoretical and analytical contributions to the field of decision science and spatial energy planning. Theoretically, it expands the scope of neutrosophic logic by applying Single-Valued Neutrosophic Sets (SVNSs) within a spatially explicit GIS-based multi-criteria framework—an approach that remains underexplored in the context of national-scale renewable energy investment. This integration allows for a more nuanced representation of expert uncertainty compared to conventional fuzzy or probabilistic models. Analytically, the model combines SVNS-based expert weighting with the VIKOR method to achieve a structured compromise solution among multiple conflicting and imprecise criteria. This hybrid mechanism improves the transparency, consistency, and adaptability of the prioritization process, especially when applied to heterogeneous regional datasets. The spatial implementation of the Priority Index (PI) within a GIS environment further enhances its operational value, enabling geographically targeted policy development. As such, the framework presented here does not merely support site selection but also advances methodological rigor in uncertainty-based spatial decision-making—thereby filling a recognized gap in both neutrosophic MCDM literature and renewable energy planning research.

## Conclusions

In alignment with Turkey’s renewable energy goals, this study developed a comprehensive decision-making framework that integrates Neutrosophic logic, the VIKOR method, and GIS-based spatial analysis to prioritize wind energy investment regions. The proposed Priority Index (PI) offers a replicable and scalable tool to guide both policymakers and investors in identifying optimal provinces for wind energy development.

The findings highlight Balıkesir, Çanakkale, and İzmir as the top three investment priority regions due to their high wind potential and existing infrastructure. Istanbul and Samsun also emerge as significant regions despite their moderate wind potential, primarily due to high energy consumption and strategic grid positioning. These results underscore the necessity of balancing wind resource availability with regional demand and grid efficiency considerations.

The study offers analytical value by combining SVNS-based expert weighting with VIKOR for compromise ranking, and theoretical value by extending neutrosophic decision-making models into national-scale GIS applications. Practically, the PI-based prioritization provides actionable insights to improve regional targeting in national policies such as YEKA and RERSM. The policy relevance is further supported by identifying underutilized but high-potential regions like Samsun, İçel, and Tekirdağ.

The Nomenclature of Territorial Units for Statistics (NUTS) is a hierarchical classification system developed by Eurostat to standardize regional divisions for statistical and planning purposes across Europe. A key limitation of the study lies in the use of NUTS-3 (provincial-level) resolution, which may obscure sub-regional variability in wind potential and land costs. Furthermore, although the PI was mapped geographically, spatial proximity between provinces was not incorporated into the modeling process. As a result, spatial autocorrelation techniques (e.g., Moran’s I) were not applicable. Future studies could address these limitations by using finer spatial units (e.g., NUTS-4 or parcel-level data) and incorporating spatial interaction effects to improve granularity and precision.

In conclusion, this study contributes a robust, uncertainty-resilient prioritization framework for renewable energy planning and lays the groundwork for more data-driven, region-specific energy strategies in Turkey and similar developing energy markets. Future research could further enhance the model by integrating dynamic policy simulations, economic feasibility analyses, and environmental impact layers to support holistic decision-making in the renewable energy sector.

## Data Availability

The datasets used and/or analysed during the current study are available from the corresponding author on reasonable request.
